# Supplementary material for: Comprehensive genomic and epigenomic analysis in cancer of unknown primary guides molecularly-informed therapies despite heterogeneity
Source: Nat Commun. 2022 Aug 2;13:4485. doi: 10.1038/s41467-022-31866-4 (PMC9346116; doi:10.1038/s41467-022-31866-4)
Supplement: Supplementary file 1 — Supplementary Information [file 41467_2022_31866_MOESM1_ESM.pdf]

Comprehensive genomic and epigenomic analysis in cancer of unknown primary guides molecularly-informed therapies despite heterogeneity

Möhrmann et al.

Supplementary Results

Alterations in *TP53* and *KRAS*

The tumor suppressor *TP53* was the most-frequently mutated gene (27 SNVs, one frameshift insertion). All *TP53* mutations were of high allele frequency (median = 0.42) and four events occurred at splice sites. Two samples had multiple *TP53* mutations: CUP-49 harbored both, a stop-gain and a nonsynonymous SNV, whereas CUP-39 harbored three distinct nonsynonymous SNVs. In all but one *TP53*-mutated samples, sCNA aberrations were identified comprising deletion and/or loss of heterozygosity (LOH). Moreover, the *TP53* region was deleted (with or without LOH) in additional eleven, and duplicated in three samples. Protooncogene *KRAS* was mutated in a total of eight samples: in seven by nonsynonymous SNVs (median allele frequency = 0.48) and in one by splice-site fusion, which was accompanied by *KRAS* overexpression. In 13 samples, including five of the ones with *KRAS* mutation, the *KRAS*-coding fragment was amplified, and in two it underwent deletion.

Genomics-based systemic treatment details

CUP-21 received pembrolizumab only during the first 2.4 months of treatment, whereas the MTBR was pembrolizumab combined with a MEK-inhibitor. CUP-39 received atezolizumab only during the first 2.3 months although the MTBR was atezolizumab combined with cobimetinib. CUP-57 was bridged with biclutamid and leuprorelin until a MTBR with higher priority (trastuzumab) was available.

Supplementary Tables

Supplementary Table 1

Supplementary Table 1.1: Detected viruses consensus

		Arriba	Kraken2					P-DiP				
		RNA	RNA	WES		WGS		RNA	WES		WGS	
**patient**	**detected virus**	**tumor**	**tumor**	**tumor**	**co**	**tumor**	**co**	**tumor**	**tumor**	**co**	**tumor**	**co**
CUP-48	Murine leukemia virus	✓	✓	N/A	N/A	✗	✗	✗	N/A	N/A	✗	✗
CUP-24	Human papillomavirus type 18	✓	✓	✓	✗	N/A	N/A	✗	✓	✗	N/A	N/A
CUP-60	Human papillomavirus type 16	✓	✓	✓	✗	N/A	N/A	✓	✓	✗	N/A	N/A
CUP-40	Torque teno virus	✓	✗	✗	✓	N/A	N/A	✗	✗	✓	N/A	N/A
CUP-02	Murine leukemia virus	✓	✓	N/A	N/A	✗	✗	✗	N/A	N/A	✗	✗
CUP-38	Human papillomavirus type 16	✓	✗	✓	✓	N/A	N/A	✗	✗	✓	N/A	N/A
CUP-70	Murine leukemia virus	✓	✗	✗	✗	N/A	N/A	✓	✗	✗	N/A	N/A
CUP-23	Human papillomavirus type 16	✓	✓	✓	✗	N/A	N/A	✓	✗	✗	N/A	N/A
CUP-06	Torque teno virus	✗	✗	N/A	N/A	✗	✓	✗	N/A	N/A	✗	✓

co = control, N/A = data type not available, ✓ = detected, ✗ = not detected

Supplementary Table 1.2: Viral integration sites

patient	sample	sample type	chromosome	position	closest gene	integrated virus	detection method
CUP-38	control	WES	7	27142069	*HOXA2*	Human papillomavirus type 16	VIRUSBreakend
CUP-24	tumor	RNA	1	67436536	*MIER1*	Human papillomavirus type 18	Arriba
CUP-24	tumor	RNA	1	67441201	*MIER1*	Human papillomavirus type 18	Arriba
CUP-24	tumor	RNA	1	67455968	*MIER1*	Human papillomavirus type 18	Arriba
CUP-24	tumor	RNA	1	67469999	*SLC35D1*	Human papillomavirus type 18	Arriba
CUP-23	tumor	RNA	11	78758764	*TENM4*	Human papillomavirus type 16	Arriba
CUP-23	tumor	RNA	11	78758781	*TENM4*	Human papillomavirus type 16	Arriba

Supplemental Figures

Suppl. Fig. 1

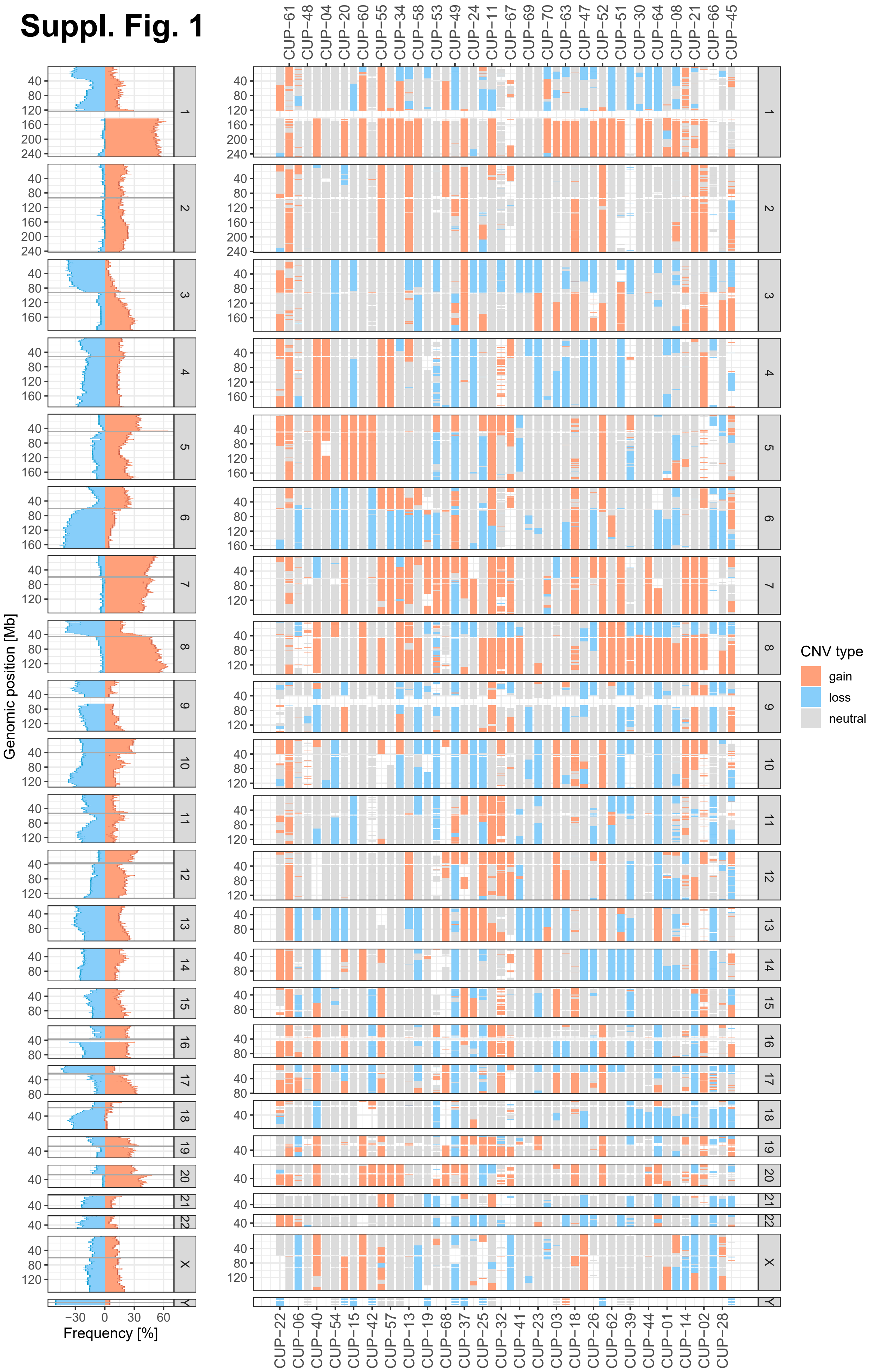

CUP−61 CUP−48 CUP−04 CUP−20 CUP−60 CUP−55 CUP−34 CUP−58 CUP−53 CUP−49 CUP−24 CUP−11 CUP−67 CUP−69 CUP−70 CUP−63 CUP−47 CUP−52 CUP−51 CUP−30 CUP−64 CUP−08 CUP−21 CUP−66 CUP−45
CUP−22 CUP−06 CUP−40 CUP−54 CUP−15 CUP−42 CUP−57 CUP−13 CUP−19 CUP−68 CUP−37 CUP−25 CUP−32 CUP−41 CUP−23 CUP−03 CUP−18 CUP−26 CUP−62 CUP−39 CUP−44 CUP−01 CUP−14 CUP−02 CUP−28
1 2 3 4 5 6 7 8 9 10 11 12 13 14 15 16 17 18 19 20 21 22 X Y
Genomic position [Mb]
Frequency [%]
−30 0 30 60
CNV type
gain
loss
neutral

Supplementary Figure S1. Somatic copy number aberrations

Amplification/deletion events (red/blue) are shown in summarized by event frequency (x axis) view in the left and expanded per sample (x axis) view in the right panel across the chromosomes (y axis). Grey horizontal lines in the left panel mark the position of centromeres. Segments with data for less than 20 patients were removed from the summary plot. The analysis identified gain and loss events, which occurred in at least 40% of samples in chromosomes 1q, 7, 8q and chromosomes 3p, 6q, 8p, 17p, respectively.

Suppl. Fig. 2

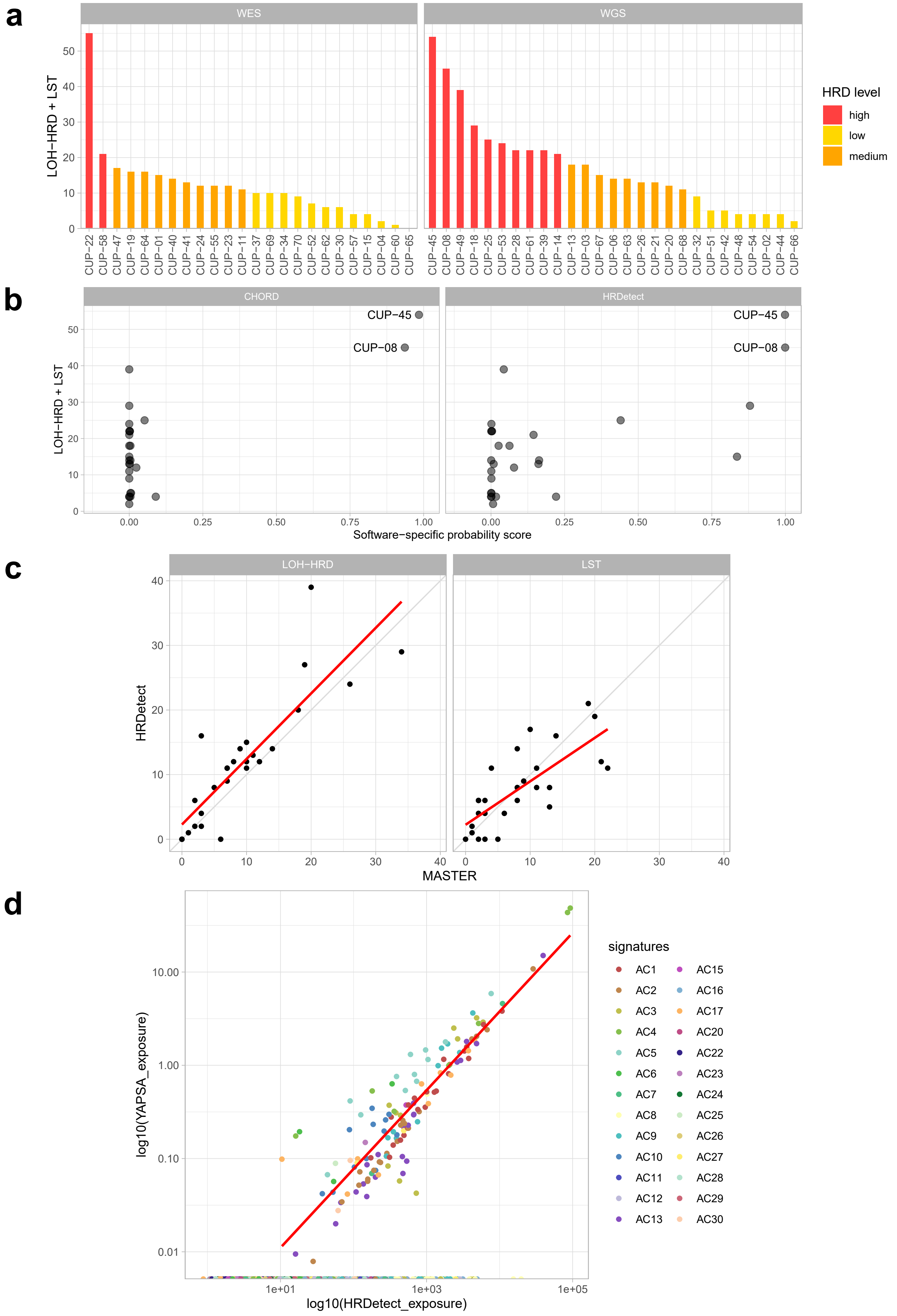
a
WES
WGS
LOH−HRD + LST
HRD level
high
low
medium
CUP−22 CUP−58 CUP−47 CUP−19 CUP−64 CUP−01 CUP−40 CUP−41 CUP−24 CUP−55 CUP−23 CUP−11 CUP−37 CUP−69 CUP−34 CUP−70 CUP−52 CUP−62 CUP−30 CUP−57 CUP−15 CUP−04 CUP−60 CUP−65
CUP−45 CUP−08 CUP−49 CUP−18 CUP−25 CUP−53 CUP−28 CUP−61 CUP−39 CUP−14 CUP−13 CUP−03 CUP−67 CUP−06 CUP−63 CUP−26 CUP−21 CUP−20 CUP−68 CUP−32 CUP−51 CUP−42 CUP−48 CUP−54 CUP−02 CUP−44 CUP−66
b
CHORD
HRDetect
CUP−45
CUP−08
LOH−HRD + LST
Software−specific probability score
c
LOH−HRD
LST
HRDetect
MASTER
d
log10(YAPSA_exposure)
log10(HRDetect_exposure)
signatures
AC1 AC2 AC3 AC4 AC5 AC6 AC7 AC8 AC9 AC10 AC11 AC12 AC13
AC15 AC16 AC17 AC20 AC22 AC23 AC24 AC25 AC26 AC27 AC28 AC29 AC30

Supplementary Figure S2. Homologous recombination deficiency

a. HRD score (sum of LOH-HRD and LST; y-axis) across all CUP samples (x-axis) stratified into two panels based on the type of sequencing data acquired. Color indicates the three levels of HR deficiency.

b. HRD score (sum of LOH-HRD and LST; y-axis) in the function of probability for HR deficiency (x-axis) calculated by CHORD (left) and HRDetect (right). Two samples, CUP-45 and CUP-08, received exceptionally high scores with all three methods used and were annotated as HR deficient and of BRCA1 type by CHORD.

c. Scatterplot of LOH-HRD (left panel) and LST (right panel) measures calculated by analysis within MASTER (x-axis) and HRDetect (y-axis). Pearson correlation coefficients are 0.86 (p = 7.7e-09) and 0.75 (p = 7.1e-06), respectively.

d. Scatterplot of exposures of COSMIC signatures in all samples calculated by HRDetect (x-axis) and YAPSA (y-axis). Pearson correlation coefficient is 0.97 (p < 2.2e-16).

Suppl. Fig. 3

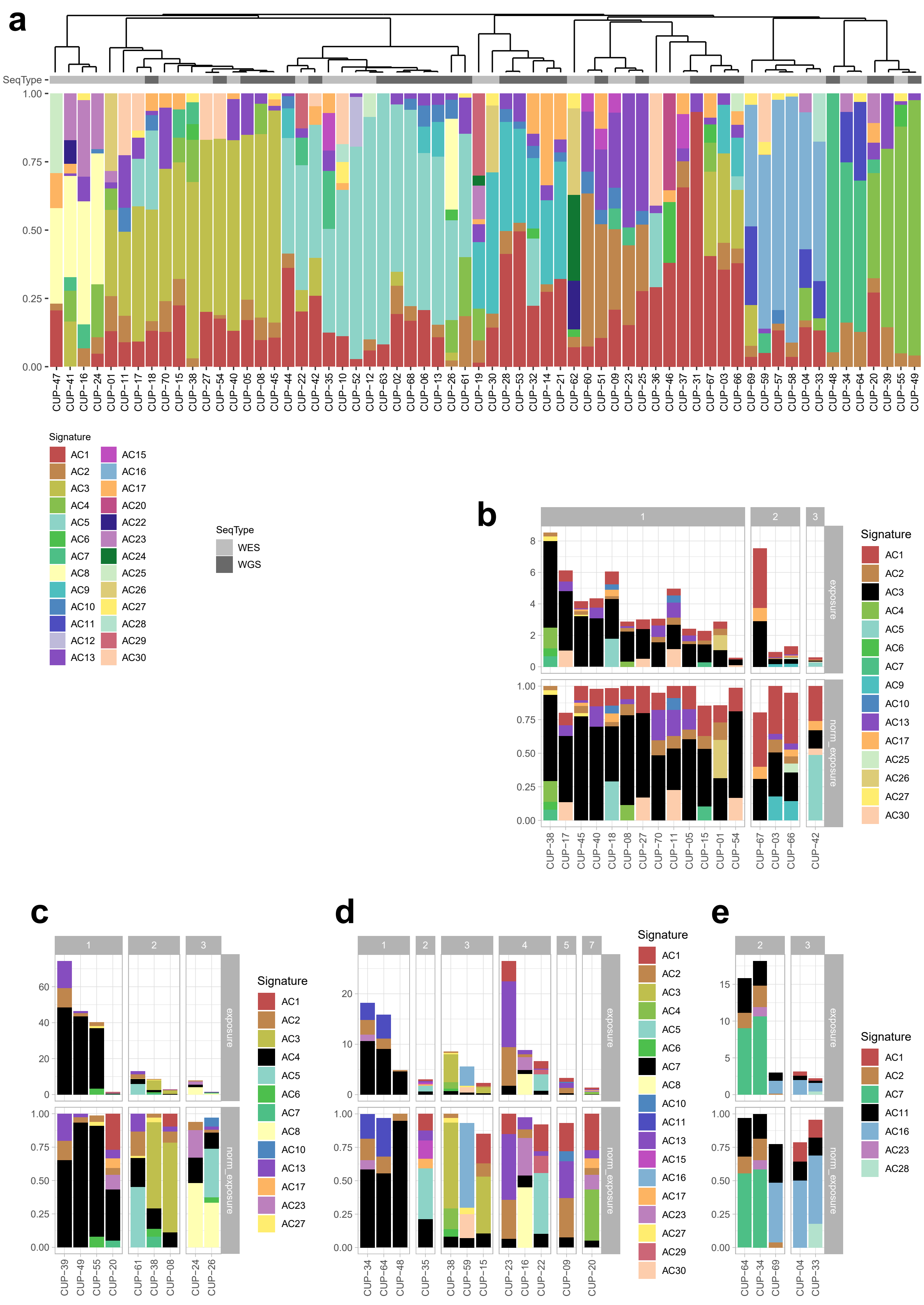
a
SeqType
1.00
0.75
0.50
0.25
0.00
Signature
AC1
AC2
AC3
AC4
AC5
AC6
AC7
AC8
AC9
AC10
AC11
AC12
AC13
AC15
AC16
AC17
AC20
AC22
AC23
AC24
AC25
AC26
AC27
AC28
AC29
AC30
SeqType
WES
WGS
b
exposure
norm_exposure
c
d
e

Supplementary Figure S3. Mutational signatures

a. Normalized exposures of COSMIC mutational signatures. Patients are ordered by hierarchical clustering.

b-e. Both unnormalized (top half) and normalized (bottom half) exposures for four notable COSMIC signatures, in samples in which the given signatures were positively identified, are shown. Vertically split panel corresponds to different ranks (numeric values), with 1 meaning that the signature was a dominant in the sample. Samples are additionally sorted by their unnormalized exposure values of the signature of interest. For clarity, the title signature of each panel is colored black in the plot.

b. Signature AC3 was present in 17 samples, all of which were not hypermutated. AC3 was the dominant signature in 13 samples. In three samples it was rank 2, and in all of them it was superseded by signature AC1.

c. Signature AC4 was present in nine samples. In tissue origin prediction based on RNA (using TCGA cohort as a reference) all four samples, in which AC4 was dominant (including all 3 hypermutated samples: CUP-39, CUP-49 and CUP-55), were identified as lung adenocarcinoma.

d. Signature AC7 was present in twelve samples, all of which were not hypermutated. In tissue origin prediction based on both methylome and transcriptome (using TCGA cohort as a reference) all 3 samples, where AC7 was dominant, were identified as either skin cutaneous melanoma (CUP-34 and CUP-64) or head and neck squamous cell carcinoma (CUP-48).

e. Signature AC11 was present in five samples, but dominant in none. In rank, it was superseded mainly by signature AC7 (CUP-64 and CUP-34) or AC16.

Suppl. Fig. 4

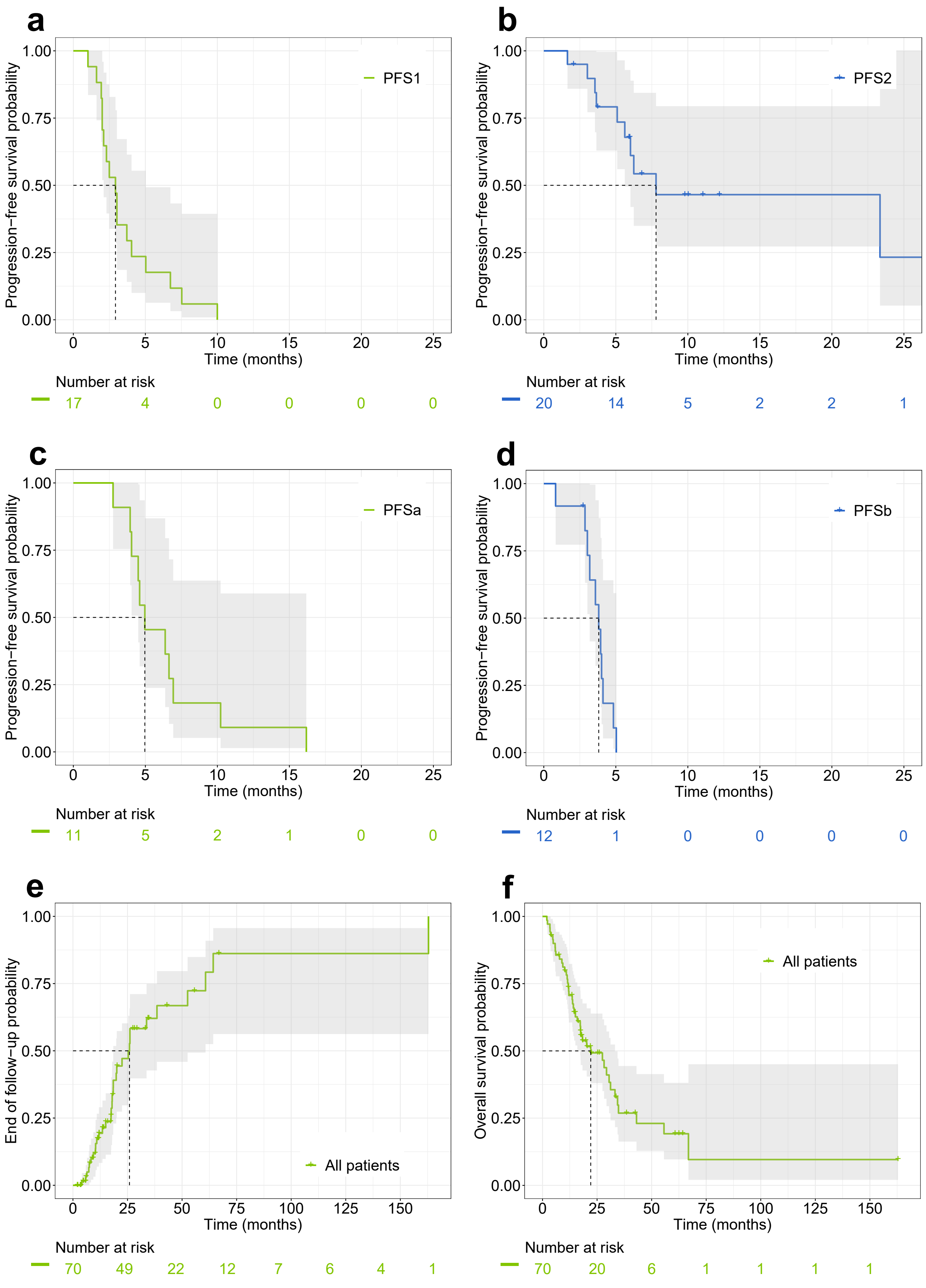
a
Progression−free survival probability
1.00
0.75
0.50
0.25
0.00
0 5 10 15 20 25
Time (months)
PFS1
Number at risk
17 4 0 0 0 0
b
Progression−free survival probability
1.00
0.75
0.50
0.25
0.00
0 5 10 15 20 25
Time (months)
PFS2
Number at risk
20 14 5 2 2 1
c
Progression−free survival probability
1.00
0.75
0.50
0.25
0.00
0 5 10 15 20 25
Time (months)
PFSa
Number at risk
11 5 2 1 0 0
d
Progression−free survival probability
1.00
0.75
0.50
0.25
0.00
0 5 10 15 20 25
Time (months)
PFSb
Number at risk
12 1 0 0 0 0
e
End of follow−up probability
1.00
0.75
0.50
0.25
0.00
0 25 50 75 100 125 150
Time (months)
All patients
Number at risk
70 49 22 12 7 6 4 1
f
Overall survival probability
1.00
0.75
0.50
0.25
0.00
0 25 50 75 100 125 150
Time (months)
All patients
Number at risk
70 20 6 1 1 1 1

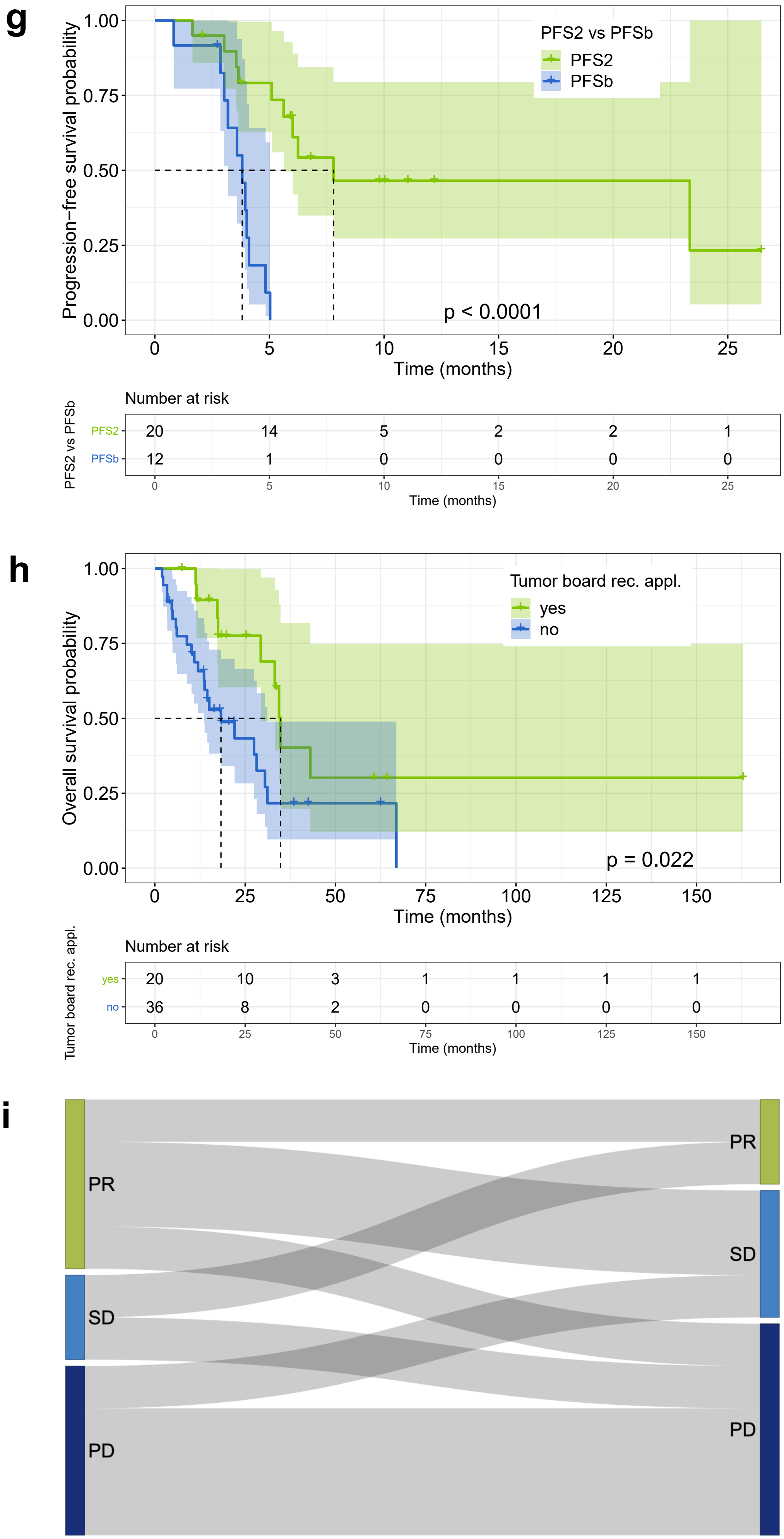

g
Progression−free survival probability
1.00
0.75
0.50
0.25
0.00
PFS2 vs PFSb
PFS2
PFSb
p < 0.0001
0
5
10
15
20
25
Time (months)
Number at risk
PFS2 vs PFSb
PFS2 20 14 5 2 2 1
PFSb 12 1 0 0 0 0
Time (months)
h
Overall survival probability
1.00
0.75
0.50
0.25
0.00
Tumor board rec. appl.
yes
no
p = 0.022
0
25
50
75
100
125
150
Time (months)
Number at risk
Tumor board rec. appl.
yes 20 10 3 1 1 1 1
no 36 8 2 0 0 0 0
Time (months)
i
PR
SD
PD
PR
SD
PD

Supplementary Figure S4. Survival analysis

Kaplan-Meier estimator plots with number at risk indicated below each graph.

a. Progression-free survival time (PFS) of the last systemic therapy prior to MASTER (PFS1, median = 2.9 months, *n* = 17). Grey area indicates 95% confidence interval.

b. PFS of the first applied treatment recommended by MASTER (PFS2, median = 7.8 months, *n* = 20). Grey area indicates 95% confidence interval.

c. PFS of the systemic therapy prior to PFSb in patients without applied recommended treatment (PFSa, median = 4.8 months, *n* = 11).

d. PFS of the first systemic therapy that was not recommended but initiated after the MTB (PFSb, median = 3.8 months, *n* = 12). Grey area indicates 95% confidence interval.

e. Follow-up time of all patients (median = 25.9 months, *n* = 70) demonstrated using the inverse Kaplan-Meier method. Grey area indicates 95% confidence intervals.

f. Overall survival of all patients (median = 22.1 months, *n* = 70).

g. Median PFSb was shorter than median PFS2 (6.0 months vs. 3.8 months, log-rank test: $p < 0.0001$). Green and blue area indicate 95% confidence intervals.

h. Median overall survival of 36 patients without application of recommended treatments was shorter than of 20 patients with applied recommended therapy (18.3 months vs. 34.8 months, log-rank test: $p = 0.022$). Green and blue areas indicate 95% confidence intervals.

rec. appl. = recommendation applied

i. Sankey plot depicting best response of therapies associated with PFSa (left) and PFSb (right). PFSa and PFSb were calculated for patients who did not receive recommended therapy. Right side depicts best response of the first systemic therapy initiated after the MTB, left side depicts best response of the last systemic therapy initiated before. Each line represents one patient, lines PR-SD and PD-PD represent two and three patients, respectively. Ten patients were available for best response comparison (CUP-03, CUP-13, CUP-37, CUP-40, CUP-44, CUP-52, CUP-54, CUP-59, CUP-63, CUP-68).

Source data are provided as a Source Data file.
