# Supplementary material for: Comprehensive genomic and epigenomic analysis in cancer of unknown primary guides molecularly-informed therapies despite heterogeneity
Source: Nat Commun. 2022 Aug 2;13:4485. doi: 10.1038/s41467-022-31866-4 (PMC9346116; doi:10.1038/s41467-022-31866-4)
Supplement: Supplementary file 3 — Description of Additional Supplementary Files [file 41467_2022_31866_MOESM3_ESM.pdf]

Description of Additional Supplementary Files

File Name: Supplementary Data 1
Description:
1.1 Basic cohort description and type of analysis
1.2 Histology
1.3 Immunohistochemistry (IHC) stainings

File Name: Supplementary Data 2
Description:
2.1 Previous therapies for patients with application of MTB recommendations
2.2 Previous therapies for patients without application of MTB recommendations

File Name: Supplementary Data 3
Description: Fusions with predictive or diagnostic relevance

File Name: Supplementary Data 4
Description:
4.1 Rare variants of unknown significance (VUS) and pathogenic / likely pathogenic germline variants (P/LP) amongst 101 cancer predisposition genes
4.2 Cancer predisposition genes that were used for rare variant filtering

File Name: Supplementary Data 5
Description: Validation cohort consisting of TCGA entities

File Name: Supplementary Data 6
Description: Entity prediction benchmarks using transcriptome data of 72 patients of the validation cohort

File Name: Supplementary Data 7
Description: Entity prediction benchmarks using methylome data of 77 patients of the validation cohort

File Name: Supplementary Data 8
Description: Comprehensive entity prediction based on methylome, transcriptome and indicative alterations

File Name: Supplementary Data 9
Description: MASTER reference cohort and respective diagnoses

File Name: Supplementary Data 10
Description:
10.1 Molecular tumor board recommendations for 56 patients
10.2 Second molecular tumor board recommendations for two patients after follow-up biopsy

File Name: Supplementary Data 11
Description: Applied recommended therapies

File Name: Supplementary Data 12

Description: PFS2/1 for 20 patients including therapy descriptions

File Name: Supplementary Data 13
Description: PFSa/b and reasons why PFSa and/or PFSb could not be calculated

File Name: Supplementary Data 14
Description: Characteristics of CUP patients with and without applied tumor board recommendations

File Name: Supplementary Data 15
Description:
15.1 Sequencing quality statistics for whole exome sequencing and kits used for DNA sample preparation
15.2 Sequencing quality statistics for whole genome sequencing and kits used for DNA sample preparation
15.3 Sequencing quality statistics for RNA sequencing and kits used for RNA sample preparation

File Name: Supplementary Data 16
Description: Top 5000 most variant CpGs used for methylation-based entity predictions

File Name: Supplementary Data 17
Description: Liver specific genes excluded in transcriptome similarity analysis

File Name: Supplementary Data 18
Description:
18.1 HRD related genes
18.2 Mutations in HRD related genes

File Name: Supplementary Software 1
Description: R script for transcriptome based entity prediction
