# Supplementary material for: Comprehensive genomic and epigenomic analysis in cancer of unknown primary guides molecularly-informed therapies despite heterogeneity
Source: Nat Commun. 2022 Aug 2;13:4485. doi: 10.1038/s41467-022-31866-4 (PMC9346116; doi:10.1038/s41467-022-31866-4)
Supplement: Supplementary file 23 — Reporting Summary [file 41467_2022_31866_MOESM23_ESM.pdf]

Corresponding author(s): Hanno Glimm

Last updated by author(s): Jun 22, 2022

# Reporting Summary

Nature Research wishes to improve the reproducibility of the work that we publish. This form provides structure for consistency and transparency in reporting. For further information on Nature Research policies, see our Editorial Policies and the Editorial Policy Checklist.

## Statistics

For all statistical analyses, confirm that the following items are present in the figure legend, table legend, main text, or Methods section.

| n/a | Confirmed | |
|---|---|---|
| ☐ | ☒ | The exact sample size (*n*) for each experimental group/condition, given as a discrete number and unit of measurement |
| ☐ | ☒ | A statement on whether measurements were taken from distinct samples or whether the same sample was measured repeatedly |
| ☐ | ☒ | The statistical test(s) used AND whether they are one- or two-sided<br>*Only common tests should be described solely by name; describe more complex techniques in the Methods section.* |
| ☒ | ☐ | A description of all covariates tested |
| ☐ | ☒ | A description of any assumptions or corrections, such as tests of normality and adjustment for multiple comparisons |
| ☐ | ☒ | A full description of the statistical parameters including central tendency (e.g. means) or other basic estimates (e.g. regression coefficient) AND variation (e.g. standard deviation) or associated estimates of uncertainty (e.g. confidence intervals) |
| ☐ | ☒ | For null hypothesis testing, the test statistic (e.g. $F$, $t$, $r$) with confidence intervals, effect sizes, degrees of freedom and $P$ value noted<br>*Give P values as exact values whenever suitable.* |
| ☒ | ☐ | For Bayesian analysis, information on the choice of priors and Markov chain Monte Carlo settings |
| ☒ | ☐ | For hierarchical and complex designs, identification of the appropriate level for tests and full reporting of outcomes |
| ☐ | ☒ | Estimates of effect sizes (e.g. Cohen's *d*, Pearson's *r*), indicating how they were calculated |

*Our web collection on statistics for biologists contains articles on many of the points above.*

## Software and code

Policy information about availability of computer code

| | |
|---|---|
| Data collection | All resources used are described in the Methods section. Patient and treatment information was documented in a centrally managed electronic data capture system (ONKOSTAR). TCGA pan-cancer methylation was retrieved via the curatedTCGAData package. FPKM expression values of the TCGA cohorts were obtained from the GDC data release v22.0. |
| Data analysis | All software used is described in the Methods section and all codes are publically available or available in Supplementary Data or Supplementary Software. Publicly available software included biobambam (version 0.0.148), Sambamba (version 0.6.5), samtools (version 0.1.19), Platypus (version 0.8.1), ANNOVAR, ACEseq (version 5.0.1), cnvKit (version 0.9.3). R (version 3.4.3), MSIsensor (version 0.2), STAR (version 2.5.1b), HRDetect, CHORD (version 2.0), YAPSA (version 1.13.3), tidyverse (version 1.2.1), ComplexHeatmap (version 1.99.5), Biobase (version 2.38.0), Kraken2 (version 2.1.2), Arriba (version 0.8 and 2.1.0), ggplot2 (version 3.3.3) and Microsoft Excel 2016. |

For manuscripts utilizing custom algorithms or software that are central to the research but not yet described in published literature, software must be made available to editors and reviewers. We strongly encourage code deposition in a community repository (e.g. GitHub). See the Nature Research guidelines for submitting code & software for further information.

## Data

Policy information about availability of data

All manuscripts must include a data availability statement. This statement should provide the following information, where applicable:

- Accession codes, unique identifiers, or web links for publicly available datasets
- A list of figures that have associated raw data
- A description of any restrictions on data availability

Genome, transcriptome and methylation data were deposited in the European Genome-phenome Archive (https://www.ebi.ac.uk/ega/datasets) under the accession number EGAS00001004786. The data are available under controlled access due to the sensitive nature of genome sequencing data, and access can be

obtained by contacting the appropriate Data Access Committee listed for each dataset in the study. Access will be granted to commercial and non-commercial parties according to patient consent forms and data transfer agreements for as long as needed. We have an institutional process in place to deal with requests for data transfer and aim for rapid response time. GENCODE (release 19) was used for gene annotation (https://www.gencodegenes.org/human/release_19.html). The raw clinical data are protected and are not available due to data privacy laws. The processed clinical data are available as Supplementary Data files. The remaining data are available within the Article, Supplementary Information, Supplementary Data or Source Data file.

# Field-specific reporting

Please select the one below that is the best fit for your research. If you are not sure, read the appropriate sections before making your selection.

- [x] Life sciences
- [ ] Behavioural & social sciences
- [ ] Ecological, evolutionary & environmental sciences

For a reference copy of the document with all sections, see nature.com/documents/nr-reporting-summary-flat.pdf

# Life sciences study design

All studies must disclose on these points even when the disclosure is negative.

| | |
|---|---|
| Sample size | Sample size was determined by tumor/control samples available. |
| Data exclusions | No data were excluded. |
| Replication | Whole-genome sequencing (WGS), whole-exome sequencing (WES), RNA sequencing and methylation analysis using the Infinium MethylationEPIC BeadChip microarray (850K) were performed once per sample (typical convention in cancer genome sequencing and personalized oncology). |
| Randomization | No randomization was performed because the study was a prospective observational study and not a randomized controlled trial. |
| Blinding | Investigators were not blinded to allocation because the study was a prospective observational study and not a randomized controlled trial. |

# Reporting for specific materials, systems and methods

We require information from authors about some types of materials, experimental systems and methods used in many studies. Here, indicate whether each material, system or method listed is relevant to your study. If you are not sure if a list item applies to your research, read the appropriate section before selecting a response.

## Materials & experimental systems

| n/a | Involved in the study | |
|---|---|---|
| x | | Antibodies |
| x | | Eukaryotic cell lines |
| x | | Palaeontology and archaeology |
| x | | Animals and other organisms |
| | x | Human research participants |
| | x | Clinical data |
| x | | Dual use research of concern |

## Methods

| n/a | Involved in the study | |
|---|---|---|
| x | | ChIP-seq |
| x | | Flow cytometry |
| x | | MRI-based neuroimaging |

# Human research participants

Policy information about studies involving human research participants

| | |
|---|---|
| Population characteristics | We performed WES or WGS of tumor tissue and matched blood from 70 patients with CUP who were enrolled in the MASTER (Molecularly Aided Stratification for Tumor Eradication Research) program, an observational study for younger adults with advanced cancer across all histologies and patients with rare tumors. Detailed clinical information is provided in Table 1. |
| Recruitment | For WES and WGS, fresh-frozen tumor specimens and matched normal control samples were collected from adult patients who had been diagnosed with CUP at 8 German cancer centers (NCT Heidelberg and Heidelberg University Hospital; NCT Dresden and University Hospital Carl Gustav Carus Dresden; West German Cancer Center, Essen; Frankfurt University Hospital, Frankfurt am Main; Charité Berlin; University of Freiburg Medical Center; University Hospital LMU Munich; University Medical Center Mainz). Samples were pseudonymized, and tumor histology and cellularity were assessed at the Institute of Pathology, Heidelberg University Hospital, prior to further processing. Our study had several potential limitations. First, our patient population was younger than one would expect for a representative CUP cohort, which can at least be partially explained by the NCT/DKTK MASTER inclusion criteria. Second, our cohort was treated with a wide range of different therapies prior to molecular analysis. Third, our study was not a randomized clinical trial but a prospective observational study. The median overall survival in our cohort was significantly longer when compared to published data which may be partially attributed to the young patient age in our cohort. |
| Ethics oversight | All patients provided written informed consent under a protocol approved by the Ethics Committee of Heidelberg University, and the study was conducted in accordance with the Declaration of Helsinki. |

Note that full information on the approval of the study protocol must also be provided in the manuscript.

# Clinical data

Policy information about clinical studies

All manuscripts should comply with the ICMJEguidelines for publication of clinical research and a completedCONSORT checklist must be included with all submissions.

| | |
|---|---|
| Clinical trial registration | *Provide the trial registration number from ClinicalTrials.gov or an equivalent agency.* |
| Study protocol | *Note where the full trial protocol can be accessed OR if not available, explain why.* |
| Data collection | The patients were enrolled 70 patients who were enrolled in MASTER between May 2013 and July 2018. Follow-up data was collected until June 2019. |
| Outcomes | Progression-free survival (PFS) of the first treatment based on MASTER (PFS2) was compared to the PFS of the last prior systemic treatment (PFS1) in each individual patient. If more than one recommended therapy was applied, PFS3 and following were calculated. The progression-free survival time ratios (PFSr) between PFS2 and PFS1 were calculated. Modified progression-free survival time ratios (mPFSr) were calculated as described by Mock et al. as cited in the manuscript. |
